# The Degradation of Automotive Radar Sensor Signals Caused by Vehicle Vibrations and Other Nonlinear Movements

**DOI:** 10.3390/s20216195

**Published:** 2020-10-30

**Authors:** Florian Hau, Florian Baumgärtner, Martin Vossiek

**Affiliations:** 1 Mercedes-Benz Cars Development, 71063 Sindelfingen, Germany; florian.baumgaertner@daimler.com; 2 Institute of Microwaves and Photonics (LHFT), Friedrich-Alexander-Universität Erlangen-Nürnberg (FAU), 91058 Erlangen, Germany; martin.vossiek@fau.de

**Keywords:** automotive radar, frequency-modulated continuous wave (FMCW) radar, nonlinear movements, phase noise, degradation, signal-to-noise ratio (SNR)

## Abstract

As the demands on modern radar systems with respect to accuracy, reliability, and availability increase, a detailed assessment of the influence of nonlinear movements has become necessary. In particular, from the point of view of radar, different types of movements, such as any kind of acceleration, braking situation, or vehicle vibration, are essential parts of any traffic scenario. These unavoidable motions, in which the relative velocity changes within one measurement cycle, are called nonlinear movements. These nonlinearities contribute to intermediate frequencies, which are comparable to the extensively described nonlinearities of a frequency ramp. This additional contribution to the intermediate signal has a direct effect on the signal-to-noise ratio and thus on the accuracy and probability of target detection. This paper presents a study of various types of nonlinear motion and a detailed definition of the resulting parameters based on a variety of vehicle-based measurements. An advanced signal model of frequency-modulated continuous wave (FMCW) radar is introduced and verified in addition to a detailed mathematical description of spectral signal behaviour in sinusoidal motions and linear acceleration. The theoretical and experimental results in idealised point targets are transferred to real complex road users. Furthermore, by applying established automotive signal processing steps in the form of an ordered statistical constant false alarm rate (OS CFAR), the consequences of determining the noise level are also shown. In combination with the already introduced signal behaviour, these results enabled general description of the signal-to-noise ratio of nonlinear movements in complex traffic scenarios.

## 1. Introduction

**Figure 1:**
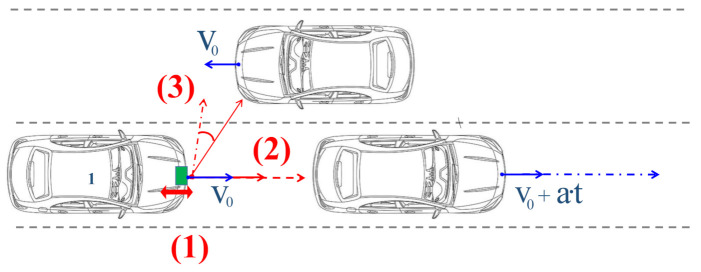
Visualization of sinusoidal movements (1), linear acceleration (2), and complex movements (3) in a daily traffic scenario.

Today, most new cars are equipped with radar-based sensors. The usage of different applications, such as safety braking functions and autonomous driving systems, have led to the prevalence of radars in automotive industries. Compared with cameras or lidar sensors, the main advantage of radar is its robustness under harsh environmental conditions [1] and the unique feature of direct and accurate speed measurement [2]. Directly determining the exact velocity characteristics of other road users is an important part of current research activities [3,4] and it enables new applications, such as pedestrian recognition [5] and enhanced object classification [6]. To meet the increasing number of requirements of new applications, the general capability to determining the velocity of other road users has increased steadily [7,8]. The main approach to realising better velocity estimation is to extend the overall measurement time. New semiconductor chip technologies have made it possible to operate higher duty cycles, which, in addition to improved signal strength because of longer integration time, has led to a direct increase in speed resolution. New applications based on small velocity effects, or so-called micro-Doppler effects, have already been established [9,10]. However, this extended measurement time and the resulting optimised velocity determination inevitably lead to a greater impact of nonlinear movements. This article provides a novel approach to determining the velocity effects based on any relative movement between the sensor and the environment. Figure 1 shows an everyday scenario in which different types of nonlinear movements or vibrations, linear accelerations, and complex accelerations are illustrated.

In general, all movements are considered linear. This assumption is based on the fact that changes in the relative velocity and the impacts of these changes are negligibly small and within one measurement cycle of a few milliseconds. To what extent this assumption is valid is discussed below. However, there are nonlinear movements in every automotive scenario. After discussing the measurement results of nonlinear movements on vehicle level in Section 2, Section 3 describes the theoretical signal deviations due to nonlinearities. In Section 4, the theoretical effects of ideal point targets are discussed in relation to real road users, such as trucks, cars, and pedestrians. The previously described signal deviations lead directly to an altered signal-to-noise ratio, which is described in Section 5 regarding typically used signal processing approaches. The materials and methods used to generate the results of vehicle measurements to radar chamber measurements are described in Section 6, and Section 7 concludes this paper.

## 2. Vehicles Measurements

**Figure 2:**
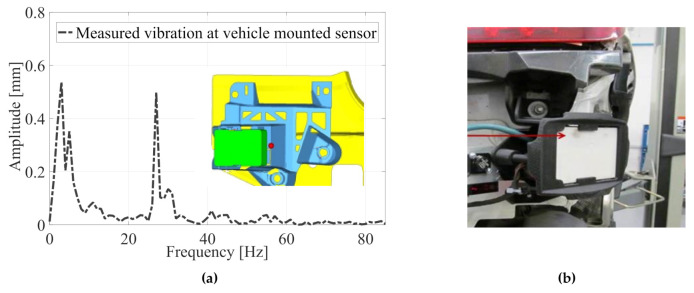
(**a**) Frequency spectrum of the vibration of an integrated radar sensor measured at the mounting system in a daily traffic situation (red dot) and (**b**) measurement setup of a body mounted sensor.

Because of the inherent dynamics of a vehicle itself, vibrations are inevitable. There are various sources of mechanical vibration, such as engine movement, air drag, and excitation due to roadway texture [11]. Because of the diversity of excitation sources and situations, the complex propagation patterns within a vehicle, and the different mounting concepts of the sensors, identifying the actual vibration that affects the radar sensors is not possible. To address this open question, the effects of excitation sources were measured in multiple vehicles directly on radar sensors in various traffic situations. These series of measurements in different vehicle settings showed that the predominant source of excitation was roadway texture. Because the resulting excitation frequency depended on a combination of the road surface and the vehicles’ speed, a very wide range of excitation frequency should be considered. The resulting vibration amplitudes are highly dependent on the natural frequencies of the mounting and nearby components of the radar and thus can vary greatly in each vehicle. The detailed series of measurements revealed that oscillations at a frequency below 100 Hz and an amplitude of 1 mm occur on a radar sensor. Strongly dominate vibrations can be closely approximated by an idealised oscillation, whereas complex oscillation patterns can be described by a summation of individual oscillations. An example of the measured nonlinear movements is illustrated in Figure 2. The vibration spectrum was measured and averaged over five seconds to emphasise the frequency-dependent movement of this particular mounting concept in the test vehicle.

Other nonlinear movements, such as acceleration and braking, are an integral part of every traffic scenario on city streets, country roads, and highways. Because the duration of a radar measuring cycle is a few milliseconds, almost every acceleration can be assumed to be constant with a good approximation. In modern advanced driver assistance systems, such as adaptive-cruise-control, accelerations and decelerations of up to $5\;\;m/s^{2}$ are realistic and values of 10 $m/s^{2}$ can be reached during emergency braking situations. For this reason, the following sections describe the effects of linear degradation on this scale.

In special situations, nonlinear movements can be either a combination of the already introduced types or even more complex, such as in a curve scenario or when the angular dependence of the relative velocity plays a major role.

## 3. Theoretical Signal Deviations Due to Nonlinear Movements

The ideal intermediate-frequency signal (IF-signal) $s_{B}\left(t\right)$ is defined in the literature by the ideal phase $\phi_{IF}\left(t\right)$. With an amplification factor *A*, a perfect signal can be described as follows:

(1) $$s_{B}\left(t\right)=A\cdot \cos\left(\phi_{\mathrm{IF}}\left(t\right)\right).$$

Of course various factors must be added to describe the beat signal in real conditions. In general, these factors are called phase noise. Many previous studies focused on the topic of phase noise in recent years, most of wich have dealt with phase noise that occurs in attempting to realise an ideal RF signal on a compact device [12,13,14,15,16]. However, [17,18,19,20,21] published theoretical descriptions of nonlinear frequency ramps and their consequences for object estimation. Furthermore, [22,23] linked these effects of phase noise to other radar sensor internal noise sources, such as thermal noise, noise in amplifiers, and noise in analogue digital conversion. In these publications, phase noise is described based on the influences of frequency ramp nonlinearities, which are referred to as $\phi_{\mathrm{nl},R}\left(t\right)$ in the present study. Moreover, another factor arises in phase based on the assumption of a linear and uniform target motion during one measurement cycle. Because in many situations the relative speed with respect to the reference system does not remain constant over the measurement period, an additional value for describing the deviation from the ideal value must be introduced. This nonlinear motion factor is referred to as $\phi_{\mathrm{nl},M}\left(t\right)$ in the following, which leads to a real beat signal:

(2) $$s_{B}\left(t\right)=A\cdot \cos\left(\phi_{\mathrm{IF}}\left(t\right)+\phi_{\mathrm{nl},R}\left(t\right)+\phi_{\mathrm{nl},M}\left(t\right)\right).$$

Because the contribution of nonlinear ramps is explained in detail in the literature [17], the present study focuses on the influence of nonlinear motion.

### 3.1. Advanced Beat Signal Model

The description of complex movements requires an extended time model for the beat signal [24]. In most cases, it is assumed that the transmitter and receiver are located at the same point in space. Based on the assumption that the runtime is determined by the distance d(t) between the sensor and the target, the speed of light *c* is given by

(3) $$\tau \left(t\right)=\frac{2d(t)}{c}\;.$$

To advance this model for application to complex movements, the accurate distance of every single target must be defined for every sample point during the entire measurement. The accurate runtime $\tau_{ch}\left(t,n\right)$ for every combination of *Tx* and *Rx* is therefore defined as the time that the HF-signal needs for the distance from the transmitting antenna to the target ${\vec{d}}_{Tx}\left(t,n\right)$ and back to the receiving antenna ${\vec{d}}_{Rx}\left(t,n\right)$:

(4) $$\tau_{ch}\left(t,n\right)=\frac{\left|{\vec{d}}_{Tx}\left(t,n\right)+{\vec{d}}_{Rx}\left(t,n\right)\right|}{c}$$

The general definition of the beat signal for one FMCW ramp can now be described in terms of centre frequency $\omega_{c}$, the amplification factor *B*, the slope factor of the frequency ramp μ, and runtime $\tau_{\mathrm{ch}}\left(t,n\right)$:

(5) $$s_{B,\mathrm{FMCW}}\left(t\right)=A\cdot \cos\left(\omega_{c}\tau_{\mathrm{ch}}\left(t\right)+\mu \tau_{\mathrm{ch}}\left(t\right)t-\frac{\mu \tau_{\mathrm{ch}}^{2}\left(t\right)}{2}\right).\;$$

Therefore, the advanced chirp-sequence FMCW(CS-FMCW) beat signal for n frequency chirps and nonlinear movements can be directly expressed as follows:

(6) $$\begin{array}{cc} s_{B,\mathrm{cs}}\left(t,n\right) & =A\cdot \cos\left(\omega \tau_{\mathrm{ch}}\left(t,n\right)+\mu \tau_{\mathrm{ch}}\left(t,n\right)-\frac{\mu \tau_{\mathrm{ch}}\left(t,n\right)}{2}\right).\;\;\; \end{array}$$

### 3.2. Signal Theories for Nonlinear Movements

In this section, the different types of nonlinear movements in automotive scenarios described in Section 1 are first described in theory before they are verified with measurements on point targets. In the case of CS-FMCW radars, the effects within one single frequency ramp are negligible because of their duration of just a few microseconds whereas the effects during the complete measurement period *T* are significant. To describe these effects, the complex phase must be defined in the range gate of a target over each individual ramp. In general, the complex phase signal $\varphi (t_{n})$ after the first fast-fourier-transformation (FFT) for a single point target is defined according to [25] by the distance dx to the target and by contributions from different noise sources. In the case of a strong target reflection, the contributions from the noise can be neglected, and therefore, the complex phase for the target range gate is directly described over *n* frequency ramps by the course of the distance between the radar and the target. For the complex phase $\varphi (t_{n})$, the following applies:

(7) $$\varphi \left(t_{n}\right)=\exp\left\{j\left(2\cdot \frac{\Delta x}{\lambda }\right)\right\}.$$

Here, $\Delta x(t_{x})$ indicates the distance and λ indicates the wavelength. For linear movements with a constant relative velocity $v_{rel}$, this yields to a well-known equation with the Doppler frequency $f_{D}$:

(8) $$\varphi \left(t_{n}\right)=\exp\left\{j\cdot 2\pi \left(2\frac{v_{rel}}{\lambda }\cdot t\right)\right\}=\exp\left\{j\pi f_{D}\cdot t\right\}.$$

The following sections describe in detail the different types of nonlinear relative movements in automotive scenarios.

#### 3.2.1. Sinusoidal Nonlinearity

Based on Equation (7) and apart from the usual linear phase term for linear movements, a periodic sensor vibration with a frequency $f_{\mathrm{vib}}$ and an amplitude $A_{\mathrm{vib}}$ causes the following additional phase-term:

(9) $$\varphi \left(t_{n}\right)=\exp\left\{ı2\pi \left(f_{D}\cdot t_{n}+2\cdot \frac{A\cdot \sin(2\pi f_{\mathrm{vib}}t_{n})}{\lambda }\right)\right\}.$$

**Figure 3:**
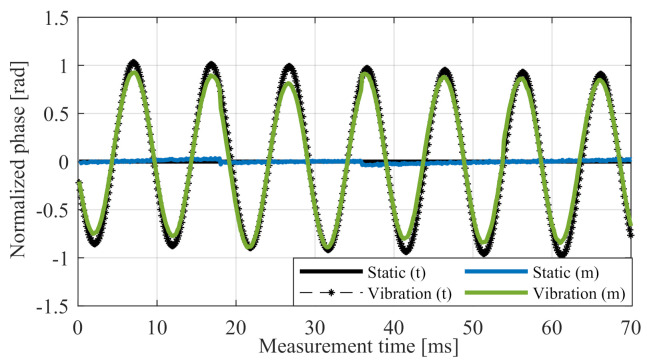
Measured phase values from the target range cell over *n* ramps for a static sensor (blue) and a vibrating sensor (green) with $f_{\mathrm{vib}}=50\;\mathrm{Hz}$ and $A_{\mathrm{vib}}=0.3\;\mathrm{mm}$, compared with the corresponding simulation (black).

Figure 3 shows the simulated phase from the advanced signal model and the measured phase for a point target with and without sensor vibrations.

The term in Equation (9) can be rewritten using the theory of phase modulation and thus by the Bessel function of the first type $J_{n}\left(z\right)$:

(10) $$\exp\left\{\hat{ız}\sin\left(\Theta \right)\right\}=\sum J_{n}\left(z\right)\exp\left\{in\Theta \right\}.$$

According to [26], the following expression results in the appropriate substitutions:

(11) $$\begin{array}{ccc} s_{\mathrm{IF}}\left(t\right) & = & \sum J_{n}\left(2\pi \frac{2\cdot A_{\mathrm{vib}}}{\lambda }\right)\;\cdot \exp\left\{ın\omega_{\mathrm{vib}}\right\}\cdot \exp\left\{ı\Theta_{\mathrm{IF}}\left(t\right)\right\}. \end{array}$$

With the second FFT over all ramps, this leads directly to

(12) $$\begin{array}{ccc} \mathrm{FFT}\left\{s_{\mathrm{IF}}\left(t\right)\right\} & = & \sum J_{n}\left(2\pi \frac{2\cdot A_{\mathrm{vib}}}{\lambda }\right)\;\cdot S_{k}\left\{\omega -nw_{\mathrm{vib}}\right\}\cdot S_{\mathrm{IF}}\left\{\Theta_{\mathrm{IF}}\left(t\right)\right\}. \end{array}$$

**Figure 4:**
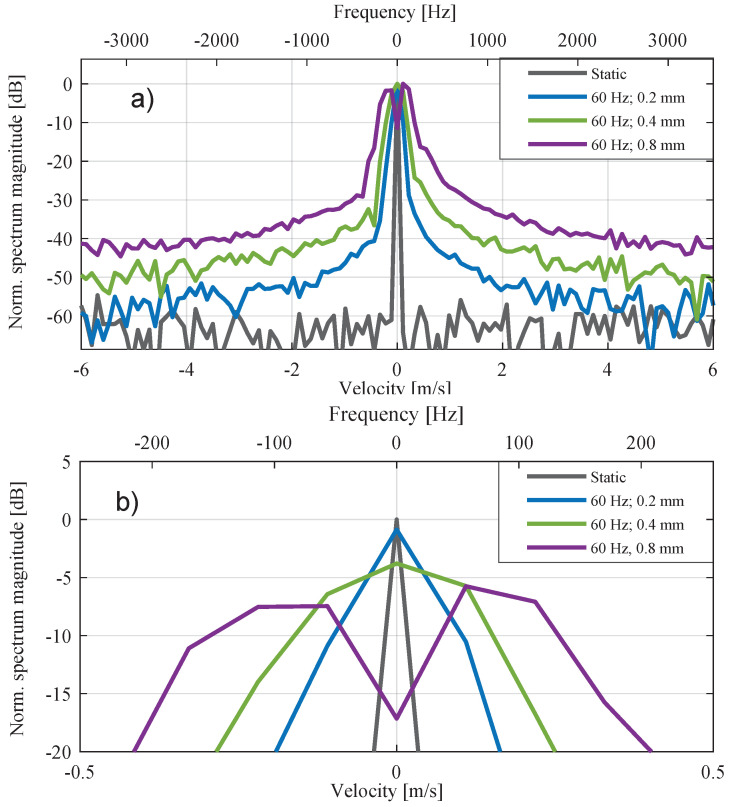
Doppler spectrum for different vibration amplitudes with additional frequency axis. (**a**) shows the complete Doppler spectrum and (**b**) the corresponding zoom out.

This equation shows that, in a radar system with wavelength λ, the influencing factor on the extent of the disorder is the amplitude of oscillation $A_{\mathrm{vib}}$. The linear term $S_{\mathrm{IF}}\left\{\Theta_{\mathrm{IF}}\left(t\right)\right\}$ defines the positions of the main peak in the Doppler spectrum while the term $S_{k}\left\{\omega -nw_{\mathrm{vib}}\right\}$ describes the minor peaks in the Doppler spectrum. Figure 4 illustrates the visible distortion of the energy density in the Doppler spectrum as the amplitude increases.

**Figure 5:**
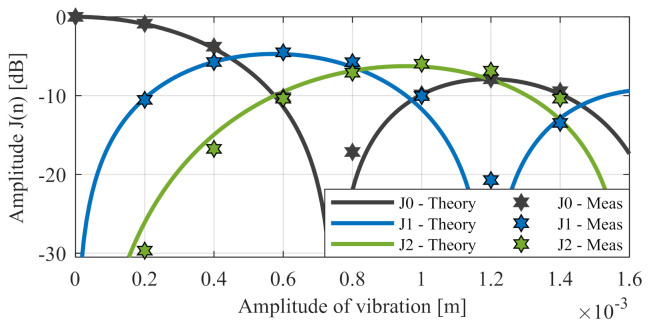
Signal level due vibration over amplitude for the main peak J${}_{0}$ and the side peaks of first order J${}_{1}$, second order J${}_{2}$, and third order J${}_{3}$.

The general signal degradation can be determined for a specific amplitude $A_{\mathrm{vib}}$ using Equation (12). Figure 5 compares the theoretical loss and the measured loss in the main peak as well as the first and second orders.

In addition to signal degradation, the broadening of the peak is an essential parameter. In phase modulation, this spectral distortion can be estimated by the Carson bandwidth $B_{C,10\%}$, which is defined as follows:

(13) $$\begin{array}{ccc} B_{C,10\%} & = & 2\cdot f_{\mathrm{vib}}\left(\eta +1\right). \end{array}$$

**Figure 6:**
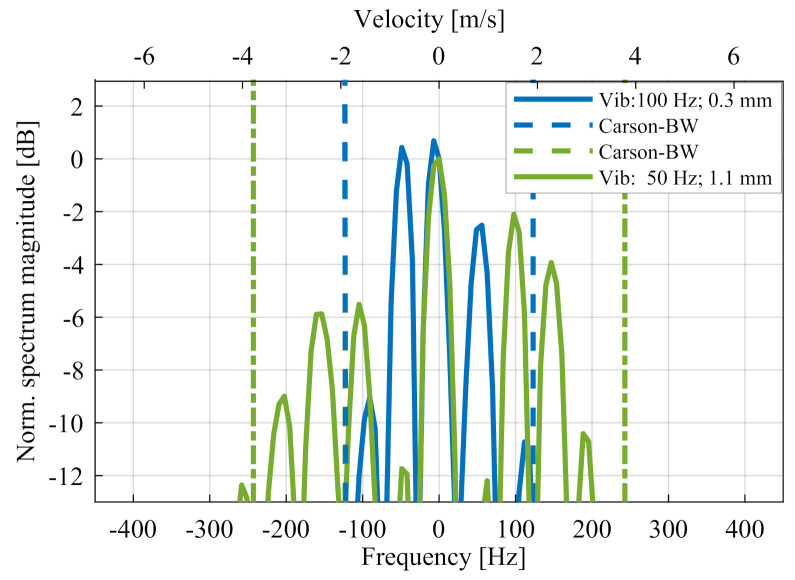
Comparison between the theoretical values for the Carson bandwidth $B_{C,10\%}$ and the measured signal behaviour of two different vibrations.

In this case, $f_{\mathrm{vib}}$ is the vibration frequency and the modulation index $\eta =2\pi \frac{2\cdot A_{\mathrm{vib}}}{\lambda }$. Figure 6 illustrates the Carson bandwidth in two different vibration amplitudes. It can be seen that the Carson bandwidth marks good approximation of the spectral range, in which signal values up to −10 dB can occur.

In the case of phase modulation, the actual signal must be modulated by complete oscillation. This leads directly to the following condition in which at least one complete oscillation is detected during measurement time $T_{n}$.

(14) $$\begin{array}{ccc} \frac{1}{T_{n}} & \leq & f_{\mathrm{vib}}. \end{array}$$

**Figure 7:**
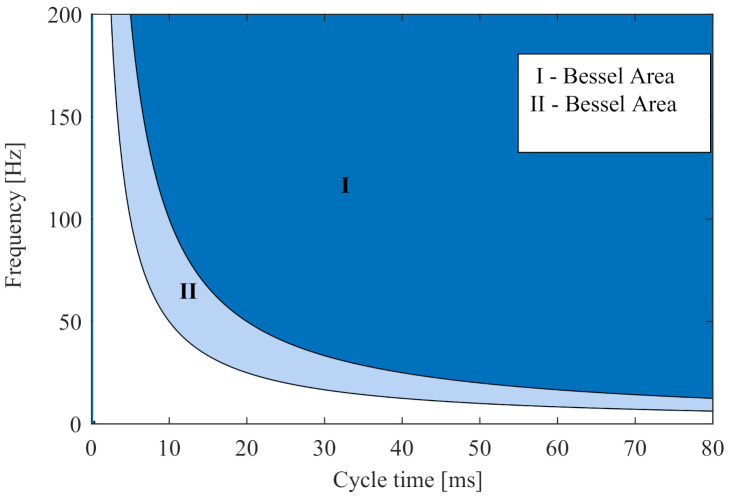
Graphical visualisation of Bessel theory scope according to vibration frequency $f_{\mathrm{vib}}$ and to overall measurement time $T_{n}$.

Combinations of $f_{\mathrm{vib}}$ and $T_{n}$ that fulfil this condition are related to the so-called Bessel area. An extensive series of measurements showed that the theory matches well with respect to vibrations as long as at least half a vibration has been completed. Therefore, in Figure 7, a second area is introduced, in which the phase modulation theory can also be applied with good approximation. The summary of the results for different vibrations are shown in Table 1.

### Constant Acceleration

In addition to the special case of vibrations described in the previous section, there are many other traffic situations in which nonlinear real-time movements can occur. Linear acceleration $a_{0}$ of any kind leads to a change in speed over measurement time *T*. From (8), we obtain the following equation for phase $\varphi_{\mathrm{lin},\mathrm{ac}}\left(t_{n}\right)$:

(15) $$\begin{array}{cc} \varphi_{\mathrm{lin},\mathrm{ac}}\left(t_{n}\right) & =\exp\left\{ı\cdot 2\pi \left(2\frac{(v_{0}+a_{0}\cdot t)\cdot t}{\lambda }\right)\right\}. \end{array}$$

Using the term for the Doppler frequency for linear movements $f_{D}=2\frac{v_{0}}{\lambda }$, the error term due to nonlinearities can be separated as follows:

(16) $$\begin{array}{cc} \varphi_{\mathrm{lin},\mathrm{ac}}\left(t_{n}\right) & =\exp\left\{ı\cdot 2\pi f_{D}t+ı\cdot 2\pi \left(2\frac{a_{0}\cdot t^{2}}{\lambda }\right)\right\}. \end{array}$$

Furthermore, this expression can be seen as an linear chirp, which is described as follows:

(17) $$\begin{array}{cc} \varphi_{\mathrm{lin},\mathrm{ac}}\left(t_{n}\right) & =\exp\left\{ı\cdot \omega_{0}t+ı\cdot \frac{\Delta \Omega }{2T}\cdot t^{2}\right\}. \end{array}$$

The spectrum of the linear chirp can be determined analytically using the Fresnel integral C(x) and S(x) in the following form:

(18) $$\begin{array}{cc} C(x) & =\int_{0}^{X}\cos\left(\frac{\pi \cdot y^{2}}{2}\right)dy;\;S\left(x\right)=\int_{0}^{X}\sin\left(\frac{\pi \cdot y^{2}}{2}\right)dy, \end{array}$$

which allows direct description of the signal behaviour. The detailed mathematical solution for the amplitude term $∥S(w)∥$ in the case of a linear frequency modulated spectrum was described in [26] as follows:

(19) $$\begin{array}{ccc} ∥S(w)∥ & = & \sqrt{\frac{\pi \Delta \Omega }{T}}{\left[{\left(C\left(X_{1}\right)+C\left(X_{2}\right)\right)}^{2}+{\left(S\left(X_{1}\right)+S\left(X_{2}\right)\right)}^{2}\right]}^{\frac{1}{2}}. \end{array}$$

The spectrum of the linear chirp can be determined analytically using the Fresnel integral C(x) and S(x) in the following form:

(20) $$\begin{array}{cc} C(x) & =\int_{0}^{X}\cos\left(\frac{\pi \cdot y^{2}}{2}\right)dy\;;\;S\left(x\right)=\int_{0}^{X}\sin\left(\frac{\pi \cdot y^{2}}{2}\right)dy, \end{array}$$

which allows direct description of the signal behaviour. In this case, the input of the Fresnel integral $X_{1}$ and $X_{2}$ is given by

(21) $$\begin{array}{ccc} X_{1} & =\frac{\frac{\Delta \Omega }{2}+\left(\omega -\omega_{0}\right)}{\sqrt{\frac{\pi \Delta \Omega }{T}}};\; & X_{2}=\frac{\frac{\Delta \Omega }{2}+\left(\omega_{0}-\omega \right)}{\sqrt{\frac{\pi \Delta \Omega }{T}}}. \end{array}$$

**Figure 8:**
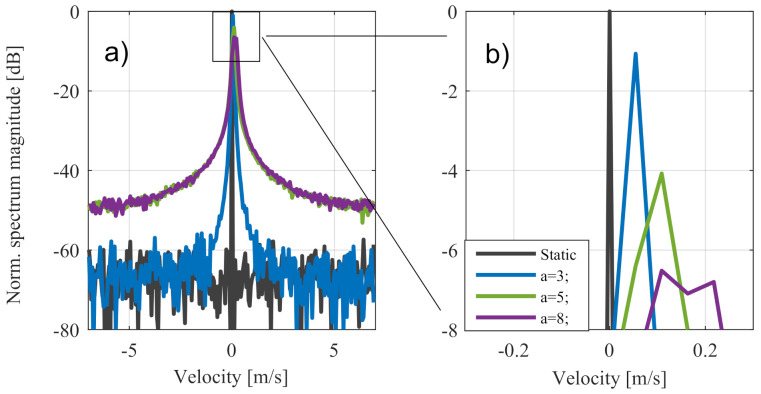
Doppler spectrum for different factors of linear acceleration compared to static result. (**a**) shows the complete Doppler spectrum and (**b**) the zoom out of the main peak.

**Figure 9:**
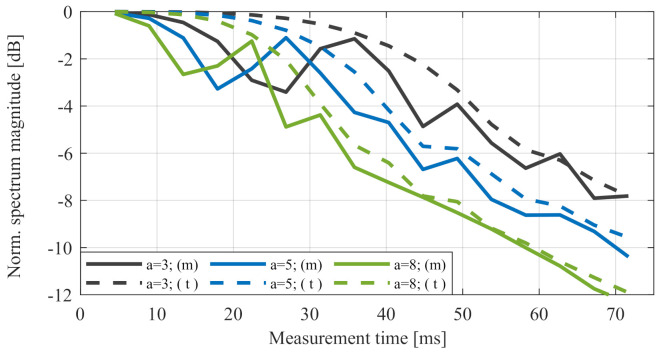
Measured signal magnitude *m* due linear acceleration over measurement time $T_{n}$ for different factors *a* compared to the theoretical values *t* according to Equation (19).

Figure 8a,b shows the measured signal degradation due to linear acceleration at the main peak in different values of acceleration $a_{0}$. Figure 9 compares measured signal degradation in different accelerations over measurement time $T_{n}$ with theoretical values in $\omega =\omega_{0}$ from (19). Here, the signal level is also normalised by the reference value with no acceleration for every timestamp of $T_{n}$. The difference between the measured and theoretical values can easily be explained by the well-known straddle effect, and it can be reduced using zero padding and window functions.

This result showed that linear acceleration had a significant drawback in high values of the overall measurement time $T_{n}$.The summary of the results for different types of accelerations are shown in Table 2.

### Complex Acceleration

**Figure 10:**
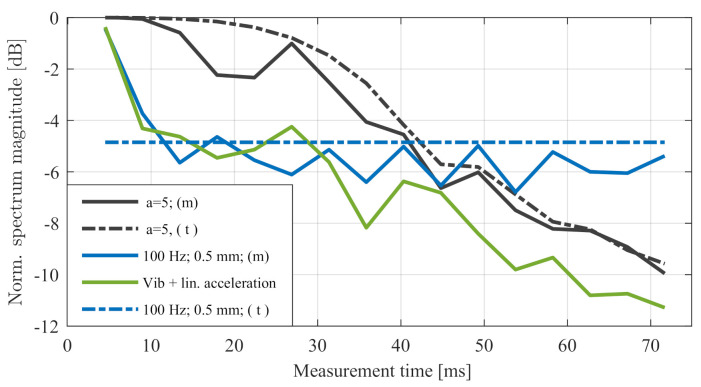
Measured signal magnitude to linear acceleration, vibration, and the combination over time compared to the theoretical values.

Accurate analysis of the movements of a real traffic scenario in detail showed that the overall relative movement was more complex than described in the previous sections. In almost every case, there was a combination of the linear movements between targets and sensors in addition to various factors, such as vibrations at different frequencies and amplitudes as well as different kinds of varying accelerations. Figure 10 illustrates the result of the combination of linear and sinusoidal accelerations within one measurement. The overall loss to complex acceleration can be described by the sum of the individual contributors. In this case, the vibration effects were predominant in cycle time $T_{n}\leq 40$ ms. In larger cycle times, the linear contribution increased in predominance.

In even more complex types of acceleration, such as any curved movement trajectory, there is no direct analytical solution. Nevertheless, the advanced signal model can be used to describe these kinds of movements for any special configuration. Because of the short measurement duration, many real movements can be approached with good approximation using an average linear acceleration.

## 4. Effects on Real Road Users

**Figure 11:**
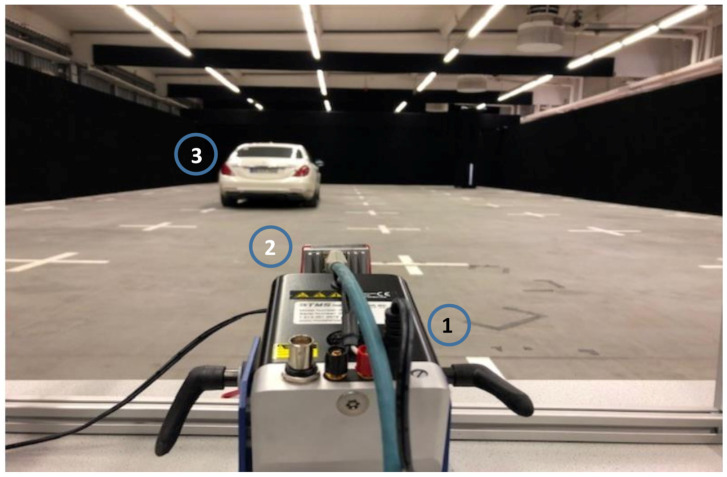
Measurement setup of a real road user in the radar chamber with the shaker system (1), the radar (2), and a car (3).

**Figure 12:**
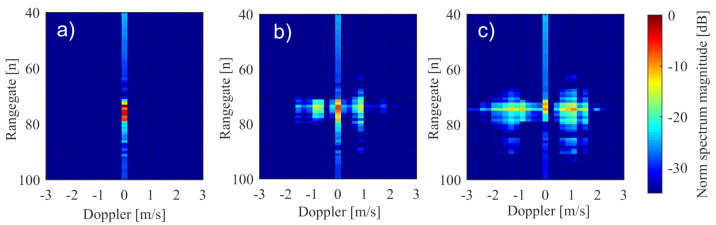
Signal behaviour for a motorcycle with a static sensor in (**a**) compared to a vibrating sensor with 50 Hz and an amplitude of A=0.1mm in (**b**) and an amplitude of A=1.2 mm in (**c**).

The previous sections described the effect of nonlinear movements on ideal point targets. In the following, these effects are analysed for typical real traffic objects such as trucks, cars, and motorcycles. Therefore, the point target reflector was replaced by different types of real road users, as shown in Figure 11. Any relative movements or vibrations were transferred from the shaker system to the sensor. The resulting ego-motion of the sensor is shown in the range-Doppler map, comparable to the behaviour of the point targets. Stretched objects, such as cars and motorcycles, have a spatial structure, which consequently spreads over more than a single range-Doppler gate. Figure 12 illustrates this with measured range-Doppler maps for motorcycle with a static sensor and a vibrating sensor.

In this case, the signal was expanded over a few range gates as expected, whereas in the Doppler direction, it fell on the Doppler gate at relative zero velocity. Figure 12b,c shows that distortion of the signal information in the Doppler direction occured when the sensor vibration increased.

**Figure 13:**
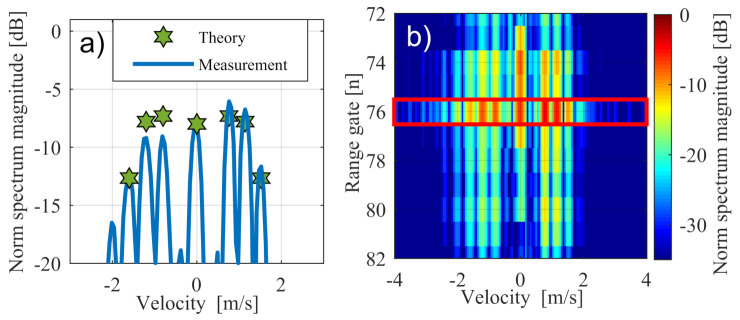
Zoom out of the Doppler spectrum in one range gate. (**a**) shows the measured Doppler spectrum in comparion with the expected theoretical values. (**b**) shows the complete Doppler spectrum (red box).

In a more precise analysis, measurement of the motorcycle in Figure 12c is shown in Figure 13 with additional zero padding up to 1024 points. The resulting Doppler spectra in the highlighted range section showed a very good match with the expected theoretical values in Section 3.

The small effect of asymmetry shown in Figure 13c can be explained by the low number of complete vibrations. The same behaviour occured analogously in other range sections of the motorcycle, where only the absolute signal level was lower.

## 5. From Signal Deviations to SNR degradation

To this point, the signal behavior has been described as influenced by nonlinear movements for ideal point targets as well as the extension to real road users. To assess the influence of nonlinearity on the probability of detection of a target and then on tracker behaviour and classification, the influence on the signal-to-noise ratio is described in this section. First, the effects of nonlinear movements are classified into noise factors reported in the literature, and then their influence is discussed by evaluating different measurements of specific targets.

### 5.1. General Contributions to SNR

In the literature, many previous studies have described SNR in an FMCW radar. For example, [23] described the interaction of different noise components, such as thermal noise $N_{\mathrm{th}}$, phase noise $N_{\mathrm{PN}}$, amplifier noise factor *F*, and the analogue to digital converter noise $N_{\mathrm{ADC}}$. Based on the assumption of uncorrelated noise sources, $N_{\mathrm{th}}$ as well as $N_{\mathrm{PN}}$ and $N_{\mathrm{ADC}}$ can be summed, whereas the factor of the amplifier *F* acts as a multiplier only in thermal noise and phase noise. With the receiving signal $S_{\mathrm{rec}}$, the following equation of $SNR_{\mathrm{FMCW}}$ is applied to one frequency ramp:

(22) $$\begin{array}{ccc} SNR_{\mathrm{FMCW}} & = & \frac{S_{\mathrm{rec}}}{\left(N_{th}+N_{\mathrm{PN}}\right)\cdot F+N_{\mathrm{ADC}}}. \end{array}$$

Obviously, the additional phase terms caused by nonlinear movements are not noise as such but representations of real movements. By reducing the maximum signal strength and the influence of the additional signal distribution in the Doppler spectrum, nonlinear movements have symptoms to compare with phase noise. Therefore, nonlinear movements can be considered a form of phase noise in comparison with the general assumption of simply being ideal linear movements with constant velocities. In contrast to the well-described forms of phase noise caused by nonlinear movements of frequency ramps, the effects of nonlinear motion cannot be determined by the single-side-band phase noise spectrum.

In chirp sequence radar, increasing SNR is expected with an increasing number *n* of equivalent FMCW ramps. Assuming the incoherent integration of individual ramps, the signal-to-noise ratio $SNR_{n,\mathrm{theory}}$ would behave as follows [27]:

(23) $$\begin{array}{cc} SNR_{n,\mathrm{theory}} & =SNR_{\mathrm{FMCW}}\cdot n^{a}. \end{array}$$

with typical values of the amplification factor 0.5≤a≤1 in incoherent integration.

### 5.2. Nonlinear Effects on Noise Level

Equation (22) shows that the exact determination of the noise level from individual constituents is complex. Therefore, as in most automotive radars, a statistical algorithm for determining the noise level should be used. In this study, a two-dimensional ordered statistic constant false alarm rate (2D OS C-FAR) was used. Hence, the following equation applies to the experimentally determined SNR after *n* frequency ramps:

(24) $$\begin{array}{cc} SNR_{n} & =\frac{S_{n}}{N_{\mathrm{OS},n}}=\frac{S_{n}}{N_{L}+N_{\mathrm{NL}}}. \end{array}$$

**Figure 14:**
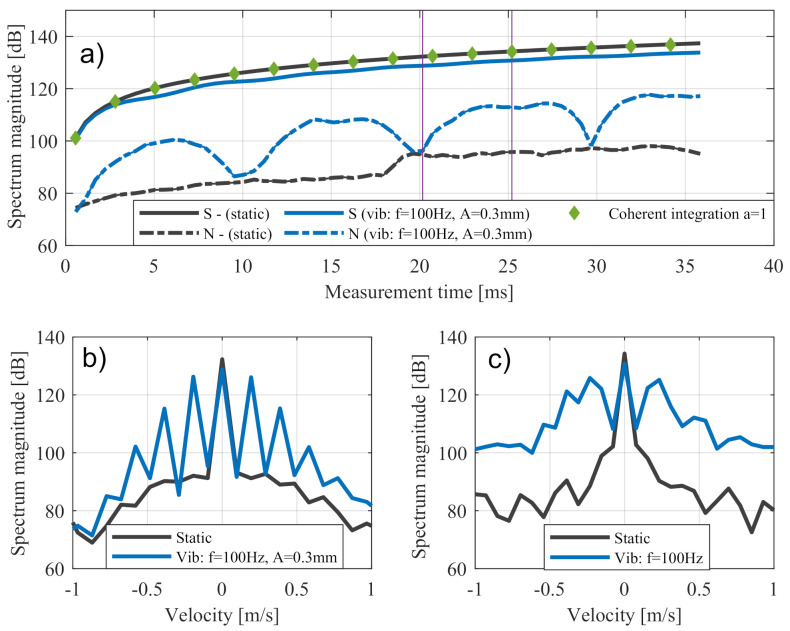
Trend for signal level and noise level over time for a static sensor and a vibrating sensor with $f_{\mathrm{vib}}=100\;\mathrm{Hz}$ and $A_{\mathrm{vib}}=0.3\;\mathrm{mm}$ in (**a**). Zoom out of the Doppler spectrum for measurement time $T_{n}=$ 20 ms in (**b**) and $T_{n}=$ 25 ms in (**c**).

As shown in Figure 14, signal level $S_{n}$ and noise level $N_{\mathrm{OS},n}$ in a sinusoidal movement are compared with a reference measurement without nonlinear movements. The signal behaviour in the static case matched the expected values from Equation (23) of an amplification factor a=1. In the nonlinear case, the expected signal degradation of 3dB, (see Figure 5) was observed because of a sensor vibration with an amplitude of 0.3mm.

It was observed that the trends in the noise levels of the two motorcycle measurements were different whereas the noise level for the case of linear movement corresponds to the expected trend. There was a disproportionately large increase in noise levels in the case of nonlinear movements. This additional increase in noise can also be explained by the broadening of the peaks, which was observed in the Doppler spectrum shown in Figure 14c. Figure 14b illustrates a special situation in which the measurement time was equal to a multiple of the oscillation period. In this case, the major maxima of the nth order were sharply emphasised whereas the proportions between the maxima assumed very small values. Therefore, the OS C-FAR values of sensor vibrations were similar to those without nonlinear effects. In general, this effect was smoothed by the use of window functions.

The general definition of the noise level is complex because it is affected by the method of calculation itself. Here, the ratio of the training and guard cells of the OS C-FAR compared with the spectral width of the nonlinearity determined the increase in noise level. In the following, the factor *V* is introduced, which was defined by the ratio of the Carson bandwidth $B_{C,10\%}$ and the spectral width of the relevant training cells in the Doppler direction $B_{\mathrm{Tc}}$ as follows:

(25) $$V=\frac{B_{C,10\%}}{B_{\mathrm{Tc}}}.$$

A rough estimation of the resulting noise level $N_{\mathrm{NL}}$ due to vibrations and other nonlinear movements can been given with the introduced correlation factor *V* in the measurements and the theoretical $SNR_{\mathrm{static}}$ value for a linear measurement:

(26) $$N_{\mathrm{NL}}≃\left(SNR_{\mathrm{static}}-10dB\right)\cdot V.$$

**Figure 15:**
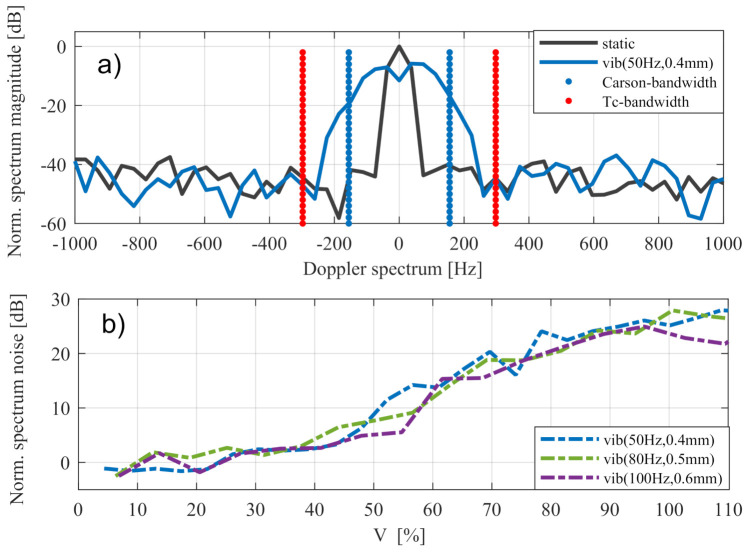
Doppler spectrum with bandwidth of training cells and Carson bandwidth in (**a**). Noise level over *V* for different vibrations in (**b**).

Figure 15 shows that additional noise in a given value of V=0<V<1 was the same in any vibration with a modulation index η>1. In values of V>1, the noise level was stagnated or decreased because parts of the widened peak passed the spectral range of the training cells $B_{\mathrm{Tc}}$.

In general, noise behaviour is strongly affected by ambient conditions, such as other road users or the infrastructure in the field of view. Therefore, these measurements of the motorcycle apply only in comparatively idealised situations with small interfering factors, and they can be considered worst-case scenarios. The effect of the increasing noise level due to nonlinear movements should be smaller in more crowded situations because of the already higher noise level.

## 6. Materials and Methods

In this section, all materials and methods used in the present study are described. The data used to descripe the movements of a vehicle-integrated radar sensor from Section 2 were recorded by three three-dimensional accelerometers, which had been directly mounted on the sensor and the bracket system. The sensor movements in the modified vehicle were analysed in different traffic scenarios over thousands of kilometres. Figure 2 shows an example of movement behaviour in one of the traffic scenarios with a rough road surface at a vehicle speed of around 80km/h. The extensive measurement results showed the complex dependency of the sensor movements on general conditions such as vehicle speed, road surface, and the structure of the vehicle. For this reason, sensor movements occur in various forms within the vehicle, which is the reason that, on one hand, detailed processing of its movements was not required and on the other hand a general description of the effects of complex movements is crucial.

**Figure 16:**
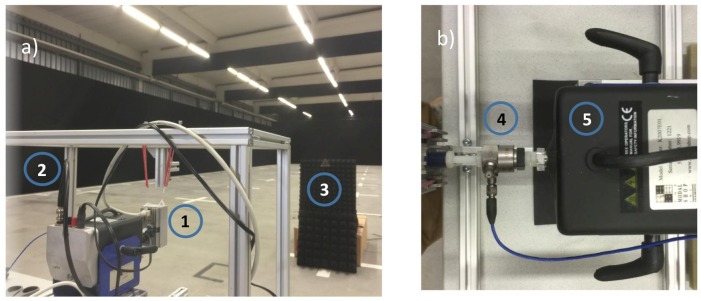
Measurement setup with the radar sensor (1), the suspension construction for the sensor (2), a corner reflector as representative of a point target (3) in (**a**). The capacity sensor to record the resulting sensor movements (4) and the shaker system (5) is shown in (**b**).

The NXP MR3003 RD Radar Transceiver Demo Kit (Böblingen, Germany) was used for all radar measurements. The raw data of the analogue-to-digital converter of the receiver channels were recorded, and the signal processing was done offline. During the radar measurements, the different reltive movements were realised by a Smart Shaker System from Synotech (Hückelhoven, Germany), which allows complex movements to be carried out controlled by an external PC, as shown in Figure 16. To precisely analyse the resulting movements initiated by the shaker system, an additional acceleration sensor was used between the sensor and the shaker system. 

## 7. Conclusions

In this study, the effects of vibration and other types of nonlinear movements on automotive FMCW radar sensors were described. For chirp sequence radar sensors, a detailed advanced signal model was developed to simulate the effects on all forms of nonlinearity. In addition, a mathematical description of sinusoidal and linear accelerations was introduced and verified by measurement on vehicle and scenario levels. Compined with the advanced signal model, both an exact determination of the signal behaviour in the presence of complex nonlinear movements and an overall classification into various criticality levels were possible dependent on the strength of the nonlinearities. These signal behaviours were not only shown as ideal point targets but also verified in a motorcycle as an example of a complex and real traffic object. In addition to signal degradation, the influence on noise level was also analysed using widely broadened signal processing steps like the 2D OS C-FAR algorithm. In these widely used processing steps, a new equation for estimating the noise level was introduced based on the spectral width of the smeared target defined by the Carson bandwidth. This provided a general description of the effects of nonlinear motion on SNRs in fast chirp FMCW radars in automotive applications. Based on this knowledge of signal behaviour due to vibrations and other nonlinear movements, the approaches to extracting movement information within a measurement cycle could be improved and the detection behaviour of individual road users could be adapted.

## Figures and Tables

**Table 1:** Categories of vibrations and their signal degradation.

Vibration Type	Frequency	Amplitude	Signal Degradation
weak vibration amplitudes	any	<0.2 mm	<2 dB
moderate vibration amplitudes	any	0.2–0.5 mm	2–5 dB
strong vibration amplitudes	any	>0.5 mm	>5 dB

**Table 2:** Categories of linear acceleration and their signal degradation

Acceleration Type	Cycle Time	Acceleration	Signal Degradation
weak acceleration	0–20 ms	<3 $\frac{m}{s^{2}}$	<2 dB
moderate acceleration	20–35 ms	3–5 $\frac{m}{s^{2}}$	2–4 dB
strong acceleration	>20 ms	>5 $\frac{m}{s^{2}}$	>4 dB

## Abbreviations

The following abbreviations are used in this manuscript:

FMCW	Frequency modulated continuous wave
SNR	Signal-to-noise ratio
OS C-FAR	Ordered statistic constant false alarm rate
